# Pulsed Vincristine Therapy in Steroid-Resistant Nephrotic Syndrome

**DOI:** 10.1155/2017/1757940

**Published:** 2017-05-29

**Authors:** Shenal Thalgahagoda, Shamali Abeyagunawardena, Heshan Jayaweera, Umeshi Ishanthika Karunadasa, Asiri Samantha Abeyagunawardena

**Affiliations:** ^1^Department of Paediatrics, Faculty of Medicine, University of Peradeniya, Kandy, Sri Lanka; ^2^Outpatient Department, Teaching Hospital Peradeniya, Peradeniya, Sri Lanka

## Abstract

Steroid-resistant nephrotic syndrome (SRNS) poses a therapeutic challenge for the paediatric nephrologist. As relentless progression to renal failure occurs with continued proteinuria, such patients will be treated with different cytotoxic medications with variable success rates and side-effects. We present here our findings on administering the anticancer drug vincristine for SRNS patients at a single centre in Sri Lanka.* Methods*. Between 2002 and 2007, fifty-four children presenting with steroid and cyclophosphamide resistance were treated with vincristine at 1.5 mg/m^2^ in weekly intravenous pulses for 8 weeks along with a tapering steroid regimen of 6 months. All patients were closely followed up for 5 years.* Results*. Of the 54 patients 39 were males and 15 were females (age range 3.5–11.6 years, median 6.1 years). At the end of the treatment course, 21 patients achieved complete remission while 7 had partial remission and no response was seen in 26 patients. Sustained remission at 6, 12, 24, and 60 months were 15 (27.78%), 11 (20.37%), 9 (16.67%), and 7 (12.96%), respectively. Most side-effects observed were reversible and no serious side-effects were noted during vincristine therapy.* Conclusion*. Although its therapeutic mechanisms in nephrotic syndrome are still not elucidated, vincristine appears to be a potent alternative that could be considered for treating SRNS.

## 1. Introduction

Nephrotic syndrome (NS) is the commonest paediatric glomerular disorder with an annual incidence of 2–7 per 100,000 [1]. While 80–90% children with NS achieve remission with initial corticosteroid therapy, the remaining 10–20% do not respond, thus being classified as steroid-resistant nephrotic syndrome (SRNS). A patient is considered to have steroid resistance if there is lack of remission despite treatment with prednisolone at a dose of 2 mg/kg/day (60 mg/m^2^/day) for 4 weeks [2]. Due to the complications of unremitting proteinuria and progressive renal disease and the side-effects of treatment with immunosuppressive medication, the management of SRNS is difficult and challenging. Failure to induce remission carries a significant risk of progression to end-stage renal disease (ESRD) within 15 years in about 50% [3].

A renal biopsy is usually undertaken in all children with SRNS before starting specific treatment. Though the renal histology of most patients with steroid sensitive nephrotic syndrome (90%) reveals minimal change nephropathy (MCN), the renal histology in SRNS is different, with up to 30–40% of patients showing focal segmental glomerulosclerosis (FSGS) [4]. The histologies in the remaining patients with SRNS include minimal change disease (30–40), mesangial proliferation, membranoproliferative glomerulonephritis, membranous nephropathy, and IgA nephropathy [2].

Children with SRNS have been treated with immunosuppressive agents such as cyclophosphamide (CYC), chlorambucil, and cyclosporine A (CYA) and more lately with mycophenolate mofetil. In those who do not respond or respond only partially, nonimmunosuppressive agents such as ACE inhibitors and angiotensin receptor blockers are employed to reduce the proteinuria.

Cyclophosphamide, although widely used in the past to treat SRNS, is now thought to have little therapeutic efficacy in the treatment of this condition. Its efficacy seems to be more in those with minimal change disease and late steroid resistance and those with a partial response to steroids. It also possesses a sinister adverse effect profile including leucopenia, haemorrhagic cystitis, reversible alopecia, gonadal toxicity, and oncological risk [5, 6]. At the time of the current study CYC was the primarily used agent for the treatment of SRNS.

CYA, a calcineurin inhibitor, has largely replaced CYC as the agent of choice for the treatment of SRNS, achieving significant complete remission rates [7]. It is effective in both minimal change disease and FSGS. This agent was however not freely available in Sri Lankan hospitals at the time of this study. Therefore other means of therapy had to be sought when patients presented with resistance to both corticosteroids and CYC. In this study we assess the efficacy of vincristine sulphate, a vinca alkaloid used in cancer therapy, in inducing and sustaining remission in SRNS.

## 2. Patients and Methods

This single-centre study was conducted at the Paediatric Nephrology Unit, Teaching Hospital Peradeniya, Sri Lanka. Children who failed to enter remission with prednisolone prescribed at a dose of 2 mg/kg/day (60 mg/m^2^/day) for 4 weeks were referred for further management. In all patients the same steroid dose was continued for additional 2 weeks during which a renal biopsy was performed. Patients who had a renal histology of either minimal change disease, idiopathic mesangial proliferation, or focal and segmental glomerulosclerosis were treated with oral cyclophosphamide (CYC) prescribed at a dose of 3 mg/Kg/day for 8 weeks along with 60 mg/m^2^ of alternate day steroids. The steroids were tapered over a period of 6 months. If remission was not achieved by 6 weeks of CYC therapy then CYC therapy was discontinued. These patients received vincristine at 1.5 mg/m^2^ in weekly intravenous pulses for 8 weeks along with a tapering course of steroids. The tapering steroid course consisted of 60 mg/m^2^ every other day for 2 weeks and then was tapered by 10 mg/m^2^ every 2 weeks over a period of 12 weeks. During therapy patients were reviewed on a weekly basis with full blood counts, urine protein excretion, serum protein and cholesterol levels, renal and liver function tests, and a full clinical examination focusing on the potential side-effects of vincristine. All possible side-effects were documented. Once they completed vincristine therapy, these patients were reviewed on a monthly basis.

We analysed the number of patients treated with vincristine from 2002 to 2007 who had complete, partial, or no remission along with the duration of sustained remission. We also analysed the adverse event profile during therapy. The collected data was entered in SPSS software version 16 and analysed using descriptive statistics and Mann–Whitney* U* test.

## 3. Results

The outcome of fifty-four children who received vincristine during this period was analysed. The ages ranged from 3.5 years to 11.6 years with a median of 6.1 years. Thirty-nine were males (72.2%) and 15 were females (27.8%). The baseline characteristics at the beginning of the study are shown in Table 1.

**Figure 1:**
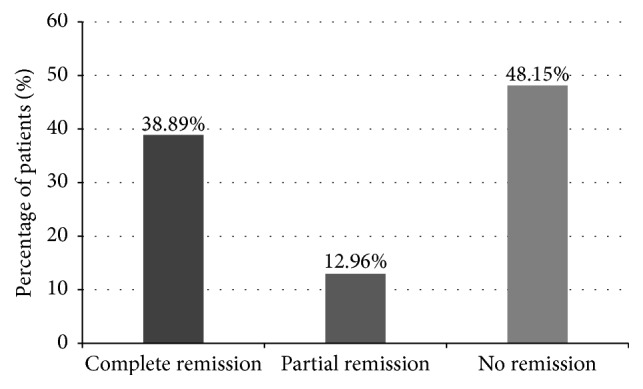
Patients achieving complete, partial, or no remission after vincristine therapy.

At the end of the course of vincristine, 21 patients out of the 54 achieved complete remission. Seven achieved partial remission and remission was not achieved in 26 patients (Figure 1).

**Figure 2:**
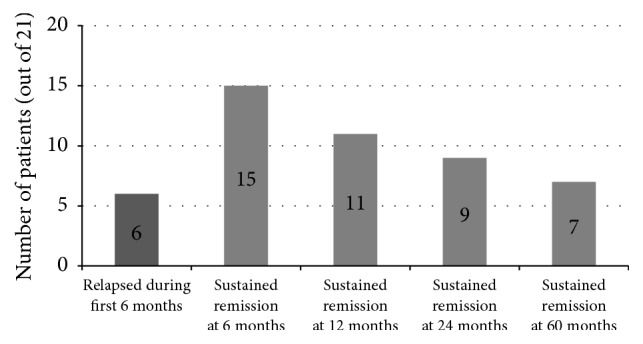
Patients who relapsed and remained in sustained remission for the first 60 months.

Out of the patients who achieved complete remission at the end of vincristine therapy, 6 (11.11%) patients relapsed during the first 6 months. Sustained remission at 6 months was seen in 15 (27.78%) patients. Eleven (20.37%), 9 (16.67%), and 7 (12.96%) patients had sustained remission at 12, 24, and 60 months, respectively (Figure 2). The number of patients having MCNS with mesangial proliferation (32/54) who achieved remission was significantly higher than that with FSGS (22/54) (*p* = 0.009).

The most frequently observed side-effects were abdominal distension and cramps, constipation, and change in the sense of taste. The occurrence of hair loss could be partly due to previous CYC therapy. None of these side-effects led to the discontinuation of treatment. The side-effects are indicated in Table 2.

## 4. Discussion

In a review of randomized controlled trials on treatment strategies in SRNS, Hodson et al. conclude that calcineurin inhibitors such as cyclosporine increase the likelihood of complete or partial remission compared with placebo/no treatment or CYC [8]. Even though we used CYC as the first-line treatment for steroid resistance, treatment options for CYC resistance were limited due to the unavailability of CYA used in developed countries for such cases. This encouraged us to use vincristine as an alternative after CYC in the present series of patients.

Vincristine is a vinca alkaloid that has played an important role as a chemotherapeutic drug for malignant diseases. It exerts antitumor activity by preventing spindle microtubule formation to disable the aligning and moving of chromosomes. Composed of two multirings, vindoline and catherantine, it interacts with *β*-tubulin at a region adjacent to the GTP-binding site known as vinca domain [9, 10]. Vincristine also is a potent inhibitor of Topoisomerase II [11].

The few previous studies done regarding vincristine and its effect on NS point towards the importance of vincristine use especially when the patient becomes resistant to steroids and second-line immunosuppressants. In 1994 Almeida et al. administered 1.5 mg/m^2^ of intravenous vincristine weekly for 8 weeks with simultaneous daily prednisolone for 4 weeks to children who were steroid resistant. With only 2 children out of 7 achieving complete remission, they concluded that their results do not encourage the use of vincristine [12]. However, Goonasekera et al. in 1998 highlighted the importance of reevaluating vincristine therapy as a potent alternative drug in patients with FSGS, based on their success with two children suffering from primary steroid- and cyclophosphamide-resistant FSGS who achieved complete remission with vincristine therapy [13]. A recent study by Kausman et al. where SDNS patients were treated with a longer regimen of vincristine reported significant reduction of relapse frequency and minimal side-effects. In addition, vincristine was also successful during subsequent relapses [14]. To add to these numbers, two children with SRNS and one child with SDNS out of 17 children achieved complete remission as reported by Krishnan et al. in 2006 [15].

The results of this study are more encouraging than the previously published literature in terms of the number of patients who achieved complete and partial remission. Most of the side-effects observed were transient and did not warrant discontinuation of the treatment. However, due to the use of steroids and CYC course prior to the vincristine therapy it is unclear if the achieved remission can be solely attributed to the action of vincristine. In spite of this, being inexpensive and reliable in patients who are noncompliant with oral medication and having fewer reversible side-effects can be stated as advantages of vincristine use [15].

How vincristine exerts its effects in nephrotic patients is still not elucidated. In an attempt to understand its action in adriamycin- (ADR-) induced nephropathy Yin et al. reported that vincristine can stabilize the actin cytoskeleton in the ADR-injured podocyte at a low dosage that does not disrupt microtubules. Their data also suggested that vincristine exerted this by suppressing overexpression of *α*3*β*1 integrin and focal adhesion kinase (FAK) [16]. This is important since inhibition of FAK activation or their deletion in podocytes has been found to protect against proteinuria and foot process effacement induced by glomerular injury [17]. Although this information provides an insight to vincristine's influence on nephropathy, further clarification of its therapeutic mechanism will definitely shed more light in this area. Thus it would be helpful to attract more attention to use it as a cheap and effective alternative treatment for NS.

## 5. Conclusions

In conclusion, it appears that vincristine is a potent and safe alternative treatment and should be considered in the treatment of SRNS. However a randomized controlled trial is required to ascertain whether it should be used in combination or as a single agent when treating SRNS.

## Figures and Tables

**Table 1:** Baseline characteristics at the beginning of the study.

Characteristics	Value
Number of patients	54
Median age (years)	6.1
Gender:	
Male	39 (72.2%)
Female	15 (27.8%)

Biopsy histology: FSGS	32 (59.3%)
MCN and mesangial proliferation	22 (40.7%)

**Table 2:** Occurrence of side-effects.

Side-effect	Number of patients
Vomiting	7
Weight loss	4
Diarrhoea	6
Bloating, abdominal pain, or cramps	21
Mouth ulcers	3
Headache	4
Hair loss	38
Constipation	13
Loss of appetite	11
Changes in sense of taste	17
Numbness and tingling in the hands and feet	8
Reversible bilateral ptosis	3
